# A Decade of Research on the Use of Three-Dimensional Virtual Worlds in Health Care: A Systematic Literature Review

**DOI:** 10.2196/jmir.3097

**Published:** 2014-02-18

**Authors:** Reza Ghanbarzadeh, Amir Hossein Ghapanchi, Michael Blumenstein, Amir Talaei-Khoei

**Affiliations:** ^1^School of Information and Communication TechnologyGriffith UniversityGold Coast, QueenslandAustralia; ^2^Agile Information Systems LabSchool of Systems, Management and LeadershipUniversity of Technology SydneySydneyAustralia; ^3^School of BusinessFaculty of Arts and BusinessUniversity of the Sunshine CoastMaroochydore DCAustralia

**Keywords:** 3D virtual worlds, 3DVW, health care

## Abstract

**Background:**

A three-dimensional virtual world (3DVW) is a computer-simulated electronic 3D virtual environment that users can explore, inhabit, communicate, and interact with via avatars, which are graphical representations of the users. Since the early 2000s, 3DVWs have emerged as a technology that has much to offer the health care sector.

**Objective:**

The purpose of this study was to characterize different application areas of various 3DVWs in health and medical context and categorize them into meaningful categories.

**Methods:**

This study employs a systematic literature review on the application areas of 3DVWs in health care. Our search resulted in 62 papers from five top-ranking scientific databases published from 1990 to 2013 that describe the use of 3DVWs for health care specific purposes. We noted a growth in the number of academic studies on the topic since 2006.

**Results:**

We found a wide range of application areas for 3DVWs in health care and classified them into the following six categories: academic education, professional education, treatment, evaluation, lifestyle, and modeling. The education category, including professional and academic education, contains the largest number of papers (n=34), of which 23 are related to the academic education category and 11 to the professional education category. Nine papers are allocated to treatment category, and 8 papers have contents related to evaluation. In 4 of the papers, the authors used 3DVWs for modeling, and 3 papers targeted lifestyle purposes. The results indicate that most of the research to date has focused on education in health care. We also found that most studies were undertaken in just two countries, the United States and the United Kingdom.

**Conclusions:**

3D virtual worlds present several innovative ways to carry out a wide variety of health-related activities. The big picture of application areas of 3DVWs presented in this review could be of value and offer insights to both the health care community and researchers.

## Introduction

Since the introduction of Web 2.0, there has been a significant improvement in the potential of Web applications. Web 2.0, as a new technology, facilitates activities such as collaboration, interaction, social networking, and participation between users [1,2]. One of the important applications of Web 2.0 is the three-dimensional virtual world (3DVW). A 3DVW is a computer-generated, simulated, networked, graphic, and multimedia environment, usually running on the Web and designed so that users can “live in” and interact via their own digital and graphical self-representations known as “avatars” [3,4]. In 3DVWs, the avatars of multiple users can interact with one another through text or voice tools, either privately or publicly, inside the various regions. By using “serious gaming” formats, avatars can move through the environments and worlds and manipulate objects. They share most of the capabilities of virtual reality (VR) technologies, especially the rendering of 3D cyberspace, they are more accessible to users via Internet-connected high-speed personal computers, and as a multi-user environment, they support social networking and interactivity.

Since the generation of 3DVWs, their lands have grown significantly with millions of residents joining globally for a variety of purposes. They offer platforms for business, education, communication, and organizational developments. As a tool for innovation, 3DVWs attract not only individuals, but also organizations, universities, companies, corporations, government agencies, and private groups that take advantage of the opportunities for collaboration, education, and communication. They are the next evolution of the Internet and social media, and they merge most of the qualities of the Web, telecommunication technology, online gaming, distance learning, social networking, creative applications, and user-generated contents.

For example, according to Linden Lab (2013), over the last 10 years, 36 million accounts have been created in Second Life (a 3DVW where users can socialize, connect, and create using voice and text chat) [5] and US $3.6 billion spent on virtual possessions. The total time users have so far spent on Second Life is equivalent to almost 217,266 years. Currently, more than a million people visit Second Life every month, and approximately 400,000 new registrations are created monthly. On average, 1.2 million daily transactions are conducted for virtual possessions, 2.1 million user-created virtual possessions are for sale, and Second Life’s landmass is nearly 700 square miles [6].

For online users, 3DVWs provide opportunities to explore, create, imagine, collaborate, role play, interact, socialize, learn, and experience events in a safe and vivid manner, and they can also be linked to the real world and other Web resources and services in a variety of scenarios. They offer improved experiences to users in relation to health care information and education, skill-building, group support, and individual consultation in terms of health. Over the last decade, there has been growing interest among the medical and public health communities in using 3DVWs for treatment, education, development, and simulation. There is a vast variety of medical and health-related 3D virtual environments inside these worlds. They currently feature a number of medical and health-related projects, have been a key area of growth, and may offer opportunities for patients, physicians, providers, educators, and health care institutions to improve both the quality and efficiency of care, treatment, and education.

Several studies have been conducted on this topic. Kamel Boulos et al introduced 3DVWs and their educational potential to medical and health educators and librarians [7]. Hansen provided an overview of 3DVWs currently used in health care professional education and medicine [8]. A survey of health-related activities on Second Life has been provided by Peck and Miller [9]. The opportunities available to nursing students within a multi-user virtual environment are presented by Beard et al [10].

The health care industry has developed tremendously due to the growing implementation of health information technology infrastructures. 3DVWs, as one of these technologies, offer great opportunities to the health community, and the health care sector are currently starting to migrate to these kinds of platforms as this technology is gradually maturing and becoming more popular and affordable. Health care organizations, universities, groups, and individuals are currently using 3DVWs for a range of clinical and health-related activities and purposes. However, the benefits of 3DVWs in health and medicine are less well understood to the health care community and researchers. It is important for them to understand the impact of 3DVWs on their field so they can consider the advantages of this technology in their own research, business, or profession. The significance of this technology in health, the remarkable attention from academia, and the lack of a comprehensive systematic literature review in this field motivated us to survey recent literature and attempt to create a big picture of 3DVWs and their application in health care. This paper attempts to answer the following three research questions:

1. What fields of health care research have been targeted by applications of 3DVW technologies?
2. In each of these fields, in what contexts have 3DVW technologies been applied?
3. What are the contributions of 3DVW technologies in each of these contexts?

## Methods

### Summary

To study the efforts made in the application of 3DVWs in the health care sector, we performed a systematic literature review, which is a methodical approach to the identification, evaluation, and interpretation of previous studies conducted on a specific research topic [11]. Our study follows the Kitchenham [11] guidelines for performing a systematic literature review. In this section, we describe the steps in the research methodology and identify the process of inclusion and exclusion of papers, as well as data extraction and analysis during the search process.

### Search and Selection Procedures

**Figure 1:**
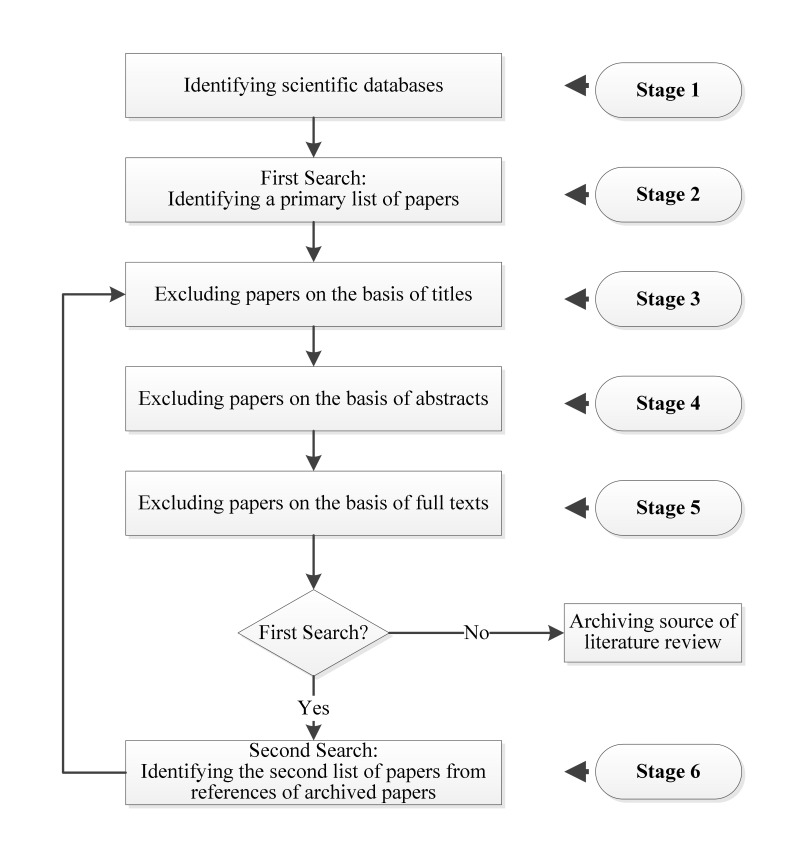
Stages in the study selection procedure.

The search process for relevant literature was completed in six stages. Figure 1 indicates the stages of study selection performed in accordance with Kitchenham [11] and Ghapanchi [12] guidelines.

In the first stage, we identified five main scientific databases in which to search for our keywords. In Stage 2, we performed a search of these five databases for 42 keywords. As of July 20, 2013, our database searches revealed 1088 primary studies. As shown in Figure 1, Stages 3 to 5 were performed twice in our search process. In the first iteration, Stage 3, we excluded 789 papers on the basis of their titles. In Stage 4, on the basis of abstracts (in some cases we had to read the full text to identify unrelated papers), 262 papers were excluded from the remaining 299 papers. The total number of remaining relevant papers was 37; however, this number of papers was not sufficient to generate an adequate conclusion. In order to increase the comprehensiveness of our study, we needed to find more papers related to this topic. According to Kitchenham [11] guidelines, one way to achieve this goal is to investigate the references of those 37 papers. Kitchenham suggests that this process can be repeated until achieving a reasonable number of papers. Therefore, we investigated the referenced papers of these 37 papers in Stage 6. This yielded an additional 1183 papers. Some 1152 papers were discarded on the basis of their titles. Another six papers were excluded because of their abstracts, resulting in 25 remaining papers. Altogether, as of August 20, 2013, we had 2271 papers, of which 2209 were discarded as being unrelated to our topic. Finally, at the end of phase two, the total number of papers was 62, which seemed reasonable for generating an appropriate conclusion. We therefore stopped the search process at phase two. Other authors have executed similar processes [12,13]. No paper was excluded in either iteration on the basis of the full text. Table 1 represents a summary of our search and the exclusion process in each stage.

**Table 1:** Summary of search and paper selection process.

	First search	References included
Initial number of papers	1088	1183
Excluded by title	789	1152
Excluded by abstract	262	6
Final number of remaining papers	37	25
Total number of remaining papers	62	

### Resources Searched

The following scientific databases were used in the search for the keywords noted in Multimedia Appendix 1: ScienceDirect, ProQuest, IEEE Explore, ACM Digital Library, and PubMed.

Table 2 shows the primary search results for the five scientific databases for searching in “all fields”, “title”, and “title/abstract/ keyword”, separately.

**Table 2:** Summary of initial search results for each database.

Database	Searched in	Number of papers found
Science Direct	Title/Abstract/Key	175
	Title	3
	All fields	13998
ProQuest	Everywhere except full text	116
	Title	0
	All fields	3119
PubMed	Title/Abstract	479
	Title	15
	All fields	760
IEEE Xplore	Abstract	261
	Title	7
	All fields	745
ACM Digital Library	Abstract	57
	Title	2
	All fields	6962

### Search Terms

The advanced search service provided by each scientific database search engine was used to perform the search operations. According to the search patterns offered by each search engine, the title, abstract, keywords, and, in some cases, the full text of articles were sourced by means of 42 search terms. Multimedia Appendix 1 shows the search terms used in each of the scientific databases. During all search processes, a publication date filtration was carried out either in the search queries or in the advanced search options.

### Inclusion and Exclusion Criteria

In order to select materials for this systematic review, some inclusion and exclusion criteria were considered. It was important for us to investigate different perspectives of applications of 3DVWs in the health care sector; therefore, we reviewed both of the design guidelines and empirical evidences for applicability in this systematic review. Accordingly, both of the empirical and technical studies have been targeted by this search. We included studies published between January 1990 and July 2013 and excluded studies in languages other than English. According to the search results, there were no relevant papers between the years 1990 and 2005.

### Data Extraction

Two types of data were extracted from 62 studies in this systematic review: (1) 3DVWs and their applications in health-related activities, and (2) year of publication, country, affiliated department, and names of sources. Consistent with health care and medical purposes, 3DVWs were designed, implemented, and applied in a majority of main categories, contexts, and subcontexts of application.

### Data Analysis

**Figure 2:**
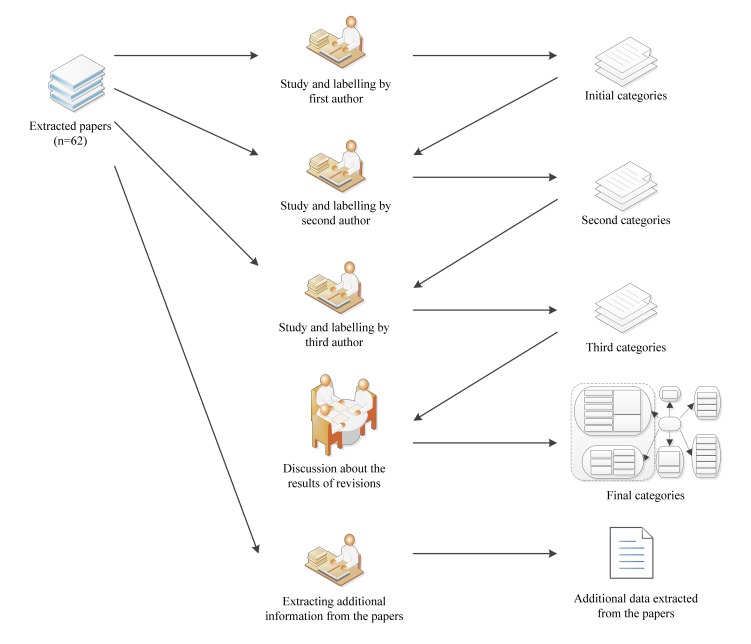
Stages in data analysis procedure.

Our purpose in this study was to group whole papers into meaningful categories of applications in health care. To perform a data analysis, we read the title, abstract, and full text of the 62 extracted papers and tried to classify each paper in an appropriate category based on focus, main area of research, and context. To accomplish this, we performed three reviews. First, each paper was reviewed completely by one of the authors and an appropriate label allocated according to the application area of 3DVWs. At the end of the first stage, all of the papers were classified in different categories. To clarify the classification, the process was repeated for the second and third times by other authors. Afterwards, the results of the first, second, and third revisions were discussed in a meeting of 5 people including the authors. During the meeting, labels were revised and some of the categories were merged. Finally, we grouped all of the extracted papers into six major research categories based on the application areas of 3DWVs. Some of the papers could have been placed in either of two different categories so we had to select the more appropriate one. After this, we attempted to extract additional information from the articles, such as contexts, subcontexts, year of publication, and country of publication. Figure 2 demonstrates the data analysis process.

## Results

### Summary

**Figure 3:**
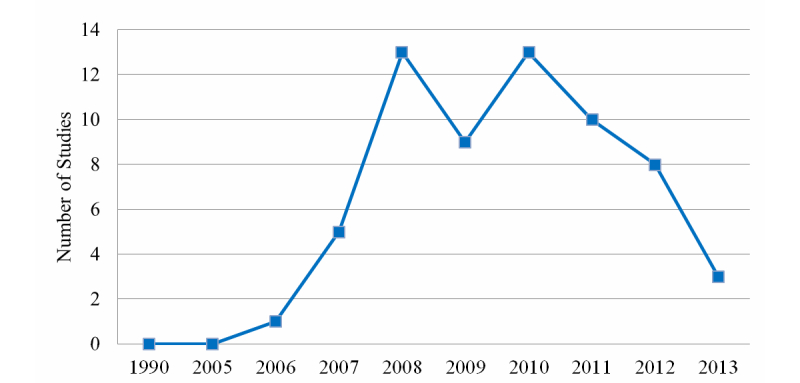
Frequency of papers per year.

Figure 3 illustrates the distribution of published papers per year. Between 1990 and 2005, there were no papers covering the application of 3DVWs in health care, primarily because 3DVWs are new technologies and the early editions of these virtual worlds were created and released in 2003. The number of published papers per year rose markedly from 1 to 13 between 2006 and 2010, decreasing to nine in 2009. There was a decline to ten papers in 2011 and eight in 2012. Our research took place in July 2013, and three papers had been published this year prior to this date.

**Figure 4:**
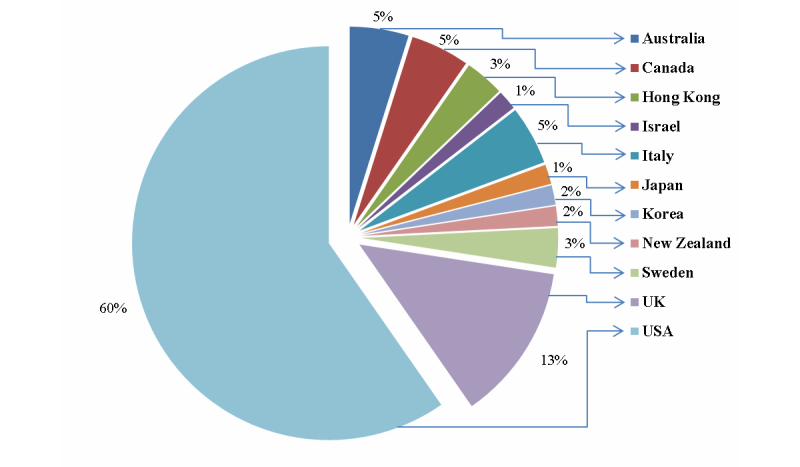
Frequency of papers from different countries.

Figure 4 shows the percentages of the extracted papers from different countries around the world. Around 60% of the total papers (37/62) were from the United States. The United Kingdom is the next source of papers in this field with 13% of publications (8/62). In third place, at 5% each (3/62), are Australia, Italy, and Canada. The remaining 12% of papers (8/62) come from Sweden, Hong Kong, Korea, Japan, Israel, and New Zealand.

Although Figure 4 does not reflect research capability, it compares papers geographically. Its main purpose is to provide insight into the amount of research done in different countries. We believe that most of the research in this field has been done in North America and may not be applicable in different health care settings, social constructs, cultural contexts, etc. Therefore, we recommend comparative research in different geographic locations.

**Figure 5:**
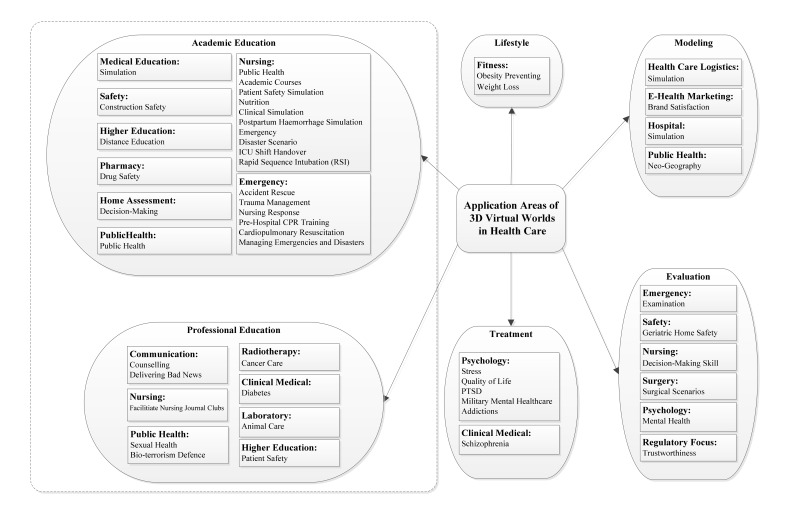
Taxonomy of application areas of 3DVWs in health care.

In order to perform a systematic review of 3DVW-related studies, we classified by field of application areas of 3DVW in health care. To provide this classification, we read all papers in our study and then attempted to assign an appropriate label to each one. In the second and third revisions, we investigated these labels and made some modifications. Finally, we found six main topics in the literature: (1) treatment, (2) modeling, (3) evaluation, (4) lifestyle, (5) academic education, and (6) professional education (see Figure 5; we grouped academic education and professional education categories using a dashed line). Academic and professional education could have been placed in a broad category of education. But to make a more accurate and detailed classification, we broke this large category to two smaller categories according to their context. The six main categories as well as their associated contexts and subcontexts are described in the following sections.

### Treatment

Studies in this category investigated different applications of 3DVWs for treatment and therapy purposes in health care. Nine studies in this category addressed the different use of these worlds in various health care treatment applications. In most cases, patients, nurses, physicians, or other medical staff had their own avatars in a specific environment in a VW, and patients were treated with specialized techniques. Treatment was performed in two health care contexts: psychological treatment and clinical treatment. For example, Gorini et al addressed the use of 3DVWs in online mental health applications and addiction treatment [14]. A method for implementing virtual environments to train people with schizophrenia to develop conversational skills in specific situations was reported by Ku et al [15]. Linton et al used Second Life to reduce stress, anxiety, and psychological distress [16].

Table 3 shows the various contexts and subcontexts related to studies for which 3DVWs were used for treatment purposes [14-22]. Most of the treatment programs were carried out on psychological and mental patients.

In order to provide meaningful insights into each application, Table 4 gives brief descriptions of each study. For example, a new approach based on using 3DVWs was described by Pioggia et al for the assessment and treatment of psychological stress [17]. A drug addiction treatment program using Second Life was introduced by Gustafson et al [22].

**Table 3:** Health care contexts and subcontexts in the treatment category.

Context	Subcontext/Reference
Psychology	Psychological stress [17]
	Quality of life of cancer patients [18]
	PTSD [19]
	Military mental health care [20]
	Stress reduction program [16]
	PTSD [21]
	Different addictions [14]
	Drug addiction [22]
Clinical/medical	Schizophrenia [15]

**Table 4:** Brief descriptions of papers in the treatment category.

Subcontext	Brief description/quote
Psychological stress	The authors describe a new approach to assessment and treatment of psychological stress based on 3DVW [17].
QoL of cancer patients	“This research investigated the effect of using a three-dimensional online chat environment on community development for cancer patients’ mental health” p. 490 [18].
PTSD	“[…a new Virtual Reality Software Suite, ARGAMAN is introduced which] is an innovative, unique solution providing an immersive virtual reality therapeutic system for treatment of PTSD (Post-Traumatic Stress Disorder) in people who witnessed or suffered terror attacks or other traumatic experiences” p. 34 [19].
Military mental health care	“…presents the design vision for a Clinical VR project called SimCoach that aims to create intelligent virtual human agents to serve the role of online health care guides/coaches for military Service Members, Veterans and their significant others in an effort to break down barriers to care” p. 504 [20].
Stress reduction program	“In this pilot study, [authors explored] the feasibility of translating a face-to-face stress reduction program into an online virtual setting and estimate the effect size of the intervention” p. 1 [16].
PTSD	“In this article, [authors] suggest that the use of a new technological paradigm, Interreality, may improve the clinical outcome of PSTV [Posttraumatic Stress Disorders]” p. 55 [21].
Different addictions	“This paper describes the development and implementation of a form of tailored immersive e-therapy called p-health whose key factor is interreality, that is, the creation of a hybrid augmented experience merging physical and virtual worlds” p. 1 [14].
Drug addiction	A drug addiction treatment program using Second Life [22]
Schizophrenia	“In this paper, [authors] report on a method of implementing virtual environments (VEs) in order to train people with schizophrenia to develop conversational skills in specific situations, which could overcome the shortcomings of or complement conventional role-playing techniques” p. 567 [15].

### Modeling

Modeling is an activity that aims to help one understand, define, quantify, visualize, or simulate a specific part or feature in the real world in a simpler way. Four studies in our systematic review examined the application of 3DVWs for modeling purposes in health care. They applied different 3DWVs for modeling of hospitals, health care logistics, eHealth marketing, and public health. In most cases, the virtual replica of a medical environment such as a university lab, hospital, hospital ward, emergency ward, or operating room were modeled and designed inside the various virtual islands using virtual worlds. The main purpose of modeling is the simulation of real-world environments for different purposes, for example, training, assessment, and examining. Kamel Boulos et al implemented a complete hospital using Second Life for several health care purposes [23]. A virtual smart hospital with tools, instruments, virtual RFID (radio-frequency identification) check, and real-time location-aware system for tracking all items was implemented by Thompson and Hagstrom using Second Life [24]. Table 5 indicates the health contexts and subcontexts of papers using 3DVWs for modeling goals [23-26].

Table 6 gives brief descriptions for each paper from the extracted studies in this category. For instance, using 3DVWs, Jin and Lee examined the effects of the regulatory fit that consumers experience in interactive eHealth marketing on their brand satisfaction and brand trust [25]. Kamel Boulos et al used Google Maps in Second Life for public health neo-geography purposes [26].

**Table 5:** Health care contexts and subcontexts in the modeling category.

Context	Subcontext/Reference
Health care logistics	Simulation [24]
eHealth marketing	Brand satisfaction and brand trust [25]
Hospital	Simulation [23]
Public health	Neo-geography [26]

**Table 6:** Brief descriptions of papers in the modeling category.

Subcontext	Brief description/quote
Simulation	A virtual smart hospital with tools, instruments, virtual RFID check, and real-time location-aware system for tracking all items [24].
Brand satisfaction and brand trust	“This study examined the effects of the regulatory fit that consumers experience in interactive e-health marketing on their brand satisfaction and brand trust” p. 673 [25].
Simulation	“This paper provides a tour of several flagship Web 3D experiences in Second Life, including Play2Train Islands (emergency preparedness training), the US Centers for Disease Control and Prevention- CDC Island (public health), Karuna Island (AIDS support and information), Tox Town at Virtual NLM Island (US National Library of Medicine - environmental health), and Jefferson’s Occupational Therapy Center” p. 290 [23].
Neo-geography	Demonstrates the use of Google maps in Second Life for public health neo-geography [26].

### Evaluation

Studies in this category focus on assessment and evaluation by 3DVWs in health care. In most cases, these worlds were used for evaluation of a particular proficiency in specific groups such as nurses and supervisors, assessment of safety in specific environments, the measurement of factors in emergency services, or investigating the rate of improvement in a patient. The main health contexts for which 3DVWs have been used for evaluation are nursing, surgery, psychology, safety regulatory focus, and emergency.

For example, Patel et al used Second Life for performance assessment of 63 surgeons in clinical scenarios [27]. In 2012, 3DVWs were used by Andrade et al to evaluate the feasibility, usability, and acceptability of virtual worlds for geriatric home safety assessments [28]. Another instance of 3DVWs’ application in evaluation in health care is the work of McCallum et al. Using qualitative evaluation, they explored students’ experiences of learning decision-making skills in the Second Life clinical simulation laboratory [29]. Table 7 shows the complete details of health care context and subcontexts of published papers related to evaluation [27-34].

Brief descriptions for each paper from the extracted papers in this category are shown in Table 8. For instance, the effects on individual performance of dyadic versus individual practice in a 3DVW home safety assessment were tested by Andrade et al [32]. Schwaab et al explored the use of Second Life virtual simulation technology to administer mock oral examinations to emergency medicine residents [34].

**Table 7:** Health care contexts and subcontexts in the evaluation category.

Context	Subcontext/Reference
Nursing	Decision-making skills [29]
Surgery	Different surgical scenarios [27]
Psychology	Mental health issues [30]
Safety	Elderly persons’ home safety assessment [31]
	Geriatric home safety simulation [32]
	Geriatric home safety [28]
Regulatory focus	Trustworthiness [33]
Emergency	Mock oral examination [34]

**Table 8:** Brief descriptions of papers in the evaluation category.

Subcontext	Brief description/quote
Decision-making skill	“… to explore nursing students’ decision-making skills through the use of a 3D virtual environment such as Second Life” p. 699 [29].
Different surgical scenarios	Second Life was used for simulating different surgical scenarios by 63 surgeons for training and assessment. [27]
Mental health issues	“This paper describes the development of a ‘rules discovery’ type game [based on Second Life] for teaching construction sequencing and the ongoing efforts to generalize the rules-discovery framework for mental health remote assessment and wider educational use” p. 14 [30].
Elderly persons’ home safety assessment	“… developed a 3-D home simulation in the virtual world Second Life containing 50 safety hazards that could affect the safety of an elderly person at home [for home safety assessment.]” p. 541 [31].
Geriatric home safety simulation	“This pilot study tests the effects on individual performance of dyadic versus individual practice in a 3D virtual world (VW) home safety assessment” p. 1 [32].
Geriatric home safety	“[Author’s] aim was to evaluate the feasibility, usability, and acceptability of virtual worlds for geriatric home safety assessments and to correlate performance efficiency in hazard identification with spatial ability, self-efficacy, cognitive load, and presence” pp. 233-234 [28].
Trustworthiness	“This study examined the influence of regulatory focus and medical recommendation avatars’ trustworthiness in avatar-based e-health within 3D virtual environments” p. 461 [33].
Mock oral examination	“In this study, [authors] explored the use of [Second Life] virtual simulation technology to administer mock oral examinations to emergency medicine residents” p. 559 [34].

### Lifestyle

In the last decade, obesity, and overweight have become a global problem. According to our studies, in order to improve people’s lifestyle, 3DVWs have been used in three different fitness programs. All three studies have used these worlds for obesity prevention and weight loss programs. For example, Johnston et al applied Second Life to implement a VW weight loss program for 54 overweight people [35]. In another study, Second Life was used by Siddiqi et al to implement an obesity prevention project [36]. See Table 9 for contexts and subcontexts of the studies using 3DVWs for lifestyle promotion [35-37].

Brief descriptions of these three papers from the extracted studies are shown in Table 10 to provide meaningful insights into each application. For instance, 3DVWs and their usefulness in obesity and diabetes therapies have been discussed by Morie and Chance [37].

**Table 9:** Health care contexts and subcontexts in the lifestyle category.

Context	Subcontext/Reference
Fitness	Obesity prevention [36]
	Obesity and diabetes/stress [37]
	Weight loss (behavioral change and self-efficacy) [35]

**Table 10:** Brief descriptions of papers in the lifestyle category.

Subcontext	Brief description
Obesity prevention	Implementation of an obesity prevention project using Second Life [36]
Obesity and diabetes/stress	How 3DVWs can be potentially powerful complements to obesity and diabetes therapies [37]
Weight loss (behavioral change and self-efficacy)	Efficacy of Second Life in a weight program relative to weight loss and behavioral change [35]

### Academic Education

It is clear that 3DVWs are an emerging medium used in both traditional classrooms and distance education. In this study, we found the majority of studies applied 3DVWs for pedagogical purposes in health care. The education category consists of two main subcategories: academic education and professional education.

Academic education focuses on academic and university-related applications of 3DVWs in health care. This category is mostly related to training programs for students and staff in universities and educational communities. Papers in this category used 3DVWs in contexts such as nursing, emergency health, and public health. For instance, in 2012, Chow et al described the development and evaluation of 3DVW for learning rapid sequence intubation (RSI) by 206 nursing students [38]. Afterwards, they explored the intention of students to use the implemented system by means of the technology acceptance model (TAM) [39,40]. Second Life has been used by the University of Michigan School of Nursing to design and implement a virtual hospital to run virtual simulations for students [41]. Veronin and colleagues’ work is another example of the use of 3DVWs in academic education. They developed an elective course at the Rangel College of Pharmacy for second- and third-year students [42]. Table 11 shows the contexts and subcontexts of the relevant papers [4,38,41-61].

Brief descriptions for each paper in the academic education category are shown in Table 12. For instance, Honey et al used Second Life for teaching postpartum hemorrhage to undergraduate nursing students and lecturers [48]. A development of virtual patient simulations for medical education using 3DVWs was performed by Danforth et al [57]. Another example of the application of 3DVWs in academic education is the work of Toth-Cohen and Gallagher [61]. They developed and evaluated a public exhibition on health and wellness at the Jefferson Occupational Therapy Education Center in Second Life.

**Table 11:** Health care contexts and subcontexts in the academic education category.

Context	Subcontext/reference
Nursing	Public health services [43]
	Graduate, undergraduate, and doctoral courses [44]
	Patient safety simulation [41]
	Nutrition [45]
	Public health issues (various activities) [46]
	Clinical simulation [47]
	Postpartum hemorrhage simulation [48]
	Acute-care medicine (emergency) [49]
	Disaster scenario [50]
	ICU first hour shift handover process [51]
	Rapid sequence intubation (RSI) [38]
Emergency	Accident rescue procedure [52]
	Trauma management [53]
	Speed and accuracy of nurse response [54]
	Pre-hospital CPR training [55]
	CPR [56]
	Managing emergencies and disasters [4]
Medical education	Virtual patient simulation [57]
Safety	Construction safety [58]
Health care higher education	Distance education [59]
Pharmacy	Drug safety [42]
Home assessment	Patient-centered decision-making [60]
Public health	Public health [61]

**Table 12:** Brief descriptions of papers in the academic education category.

Subcontext	Brief description/quote
Public health services	“…the authors describe how Second Life was integrated into a community nursing course” p. 74 [43].
Graduate, undergraduate, and doctoral courses	Second Life, as a 3DVW, found to be an environment that can provide valuable educational experiences in nursing [44].
Patient safety simulation	“The purpose of this article is to discuss how the University of Michigan School of Nursing designed and implemented a virtual hospital unit in Second Life to run virtual simulations” [41] p. 469.
Nutrition	Use of Second Life to teach interview skills [45].
Public health issues (various activities)	(1) Using Second Life in an online BSN program, (2) providing clinical experiences not often encountered, (3) using technology to help students feel connected to their classmates and instructors [46].
Clinical simulation	“… to explore Second Life as a clinical simulation platform, based on the attitudes and experiences of a sample of undergraduate nursing students” p. 883 [47].
Postpartum hemorrhage simulation	Use of Second Life for teaching postpartum hemorrhage to undergraduate nursing students and lecturers from New Zealand and the United States [48].
Acute-care medicine (emergency)	“[Authors] present three virtual world studies for team training and assessment in acute-care medicine: (1) training emergency department (ED) teams to manage individual trauma cases; (2) prehospital and in-hospital disaster preparedness training; (3) training ED and hospital staff to manage mass casualties after chemical, biological, radiological, nuclear, or explosive incidents” p. 161 [49].
Disaster scenario	“[Second Life]was implemented into an accelerated online nursing program” p. 152 [50].
ICU first hour shift handover process	“…aim was to design and develop a novel virtual world application for teaching and training Intensive Care nurses in the approach and method for shift handover, to provide an independent, but rigorous approach to teaching these important skills” p. 178 [51].
Rapid sequence intubation (RSI)	“…describes the development and evaluation of a virtual environment, the online 3D world Second Life (SL), for learning rapid sequence intubation (RSI)” p. 1136 [38].
Accident rescue procedure	“…describe experience developing virtual world-based training systems for two health care contexts. In one, procedural training was emphasised, while the other focused on teaching communication skills” p. 89 [52].
Trauma management	“…describes a project to develop and evaluate a computer-based simulator (the Virtual Emergency Department) for distance training in teamwork and leadership in trauma management” p. 321 [53].
Speed and accuracy of nurse response	“…explores immersive virtual reality as a potential educational strategy for nursing education and describes a project to develop and pioneer its use” p. 314 [54].
Pre-hospital CPR training	“…report on a study that investigates the relationship between repeated training of teams managing a medical emergency (CPR) in a Virtual World and performance outcome measures in a group of 12 medical students” p. 89 [55].
Cardiopulmonary resuscitation (CPR)	“In addition to finding a feasible way to implement CPR training, authors’ aim was to investigate how a serious game setting in a virtual world using avatars would influence medical students’ subjective experiences as well as their retention of knowledge” p. 1 [56].
Managing emergencies and disasters	“…explore the geo-data display potential of virtual worlds and their likely convergence with mirror worlds in the context of the future 3-D Internet or Metaverse, and reflect on the potential of such technologies and their future possibilities, eg, their use to develop emergency/public health virtual situation rooms to effectively manage emergencies and disasters in real time” p. 1 [4].
Virtual patient simulation	“the development of virtual patient simulations for medical education. In order to simulate real patients with greatest fidelity, the virtual patients [were] controlled by artificial intelligence” p. 3 [57].
Construction safety	“This paper proposes the adoption of online 3D world Second Life (SL) platform which allows students to perform role-playing, dialogic learning, and social interaction for efficient and effective construction safety and health education” p. 1 [58].
Distance education	Distance learning program in health care higher education using Second Life [59].
Drug safety	“…an elective course at the Rangel College of Pharmacy in pharmacy case studies for second- and third-year Doctor of Pharmacy students using Second Life” p. 105 [42].
Patient-centered decision-making	“The purpose of this research was to better understand the utility of a Web-based virtual environment as a teaching tool to represent clinical assessment and interventions in the home environment” p. 199 [60].
Public health	“…the development and evaluation of public exhibits on health and wellness at the Jefferson Occupational Therapy Education Center in Second Life” p. 3 [61].

### Professional Education

The second category of education is professional education. In this category, researchers used 3DVWs for training in professional health care such as training programs for nurses, physicians, hospital staff, and so on, in health care contexts such as nursing, public health, radiotherapy, and clinical medicine. All papers in this group conducted educational programs for non-academic learners. For example, Andrade et al studied the feasibility of using 3DVW in training medical trainees to deliver bad news to patients [62]. Kamel Boulos designed a sexual health project in Second Life to provide education about sexually transmitted infections, unintended pregnancy, and improvements in sexual relationships [63,64]. Table 13 shows the professional education category of 3DVWs applied in various health care contexts and subcontexts [62-72].

Brief descriptions for each paper in the professional education category are shown in Table 14. For example, the usage of Second Life in health care education and its ability to improve patient safety were explored by Lee and Berge [69]. Watson et al presented a framework that demonstrates how 3DVWs can be applied to meet the needs of patients with diabetes [70].

**Table 13:** Health care contexts and subcontexts in the professional education category.

Context	Subcontext/Reference
Radiotherapy	Cancer care [66]
Nursing	Facilitate nursing journal clubs [67]
Patient-centered communication	Counseling patients about colorectal cancer [68]
	Delivering bad news to patients [62]
Health care higher education	Patient safety [69]
Clinical medical	Diabetes care [70]
	Diabetes type 2 [71]
Laboratory	Animal care [72]
Public health	Sexual health [63,64]
	Bio-terrorism defence event [65]

**Table 14:** Brief descriptions of papers in the professional education category.

Subcontext	Brief description/quote
Cancer care	“[authors] propose a novel Web based e-learning application design approach that uses Discrete Event System Specification (DEVS) formalism to form and model the 3D virtual hospital Web based application for radiotherapy and cancer care treatment class of applications” p. 1 [66].
Facilitate nursing journal clubs	“The purpose of this mixed-methods pilot study was to explore the feasibility of using Second Life to conduct research and to describe nurses’ experiences in using Second Life to facilitate nursing journal clubs” p. 146 [67].
Counseling patients about colorectal cancer	“…aims were to (1) explore the feasibility, acceptability, and effectiveness of a virtual-world platform for delivering MI training designed for physicians and (2) pilot test instructional designs using SL for MI training” p. 77 [68].
Delivering bad news to patients	8 medical trainees viewed an avatar-mediated training in Second Life as an instructional method for learning how to deliver bad news to patients [62].
Patient safety	“…the usage of Second Life in health care education and its ability to improve patient safety” p. 17 [69].
Diabetes care	“…a framework that demonstrates how applications within SL can be constructed to meet the needs of patients with diabetes, allowing them to attend group visits, learn more about lifestyle changes, and foster a sense of support and emotional well-being” p. 697 [70].
Diabetes Type 2	“a pilot postgraduate medical education program in the virtual world, Second Life” p. 1 [71].
Animal care	“The article explores the potential utility of multi-user virtual environments for advancing laboratory animal care and use through better education and training” p. 163 [72].
Sexual health	“…an evaluation of a sexual health project in Second Life, designed to provide education about sexually transmitted infections, prevention of unintended pregnancy and promotion of equalitarian sexual relationships” p. 279 [63].
	“…the University of Plymouth Sexual Health SIM in Second Life… and provides some reflections on its design, as well as some details about the planned evaluation of the project” p. 1 [64].
Bio-terrorism defence event	Creation and evaluation of a pilot bioterrorism defence training environment using virtual reality technology [65].

## Discussion

### Principal Findings

**Figure 6:**
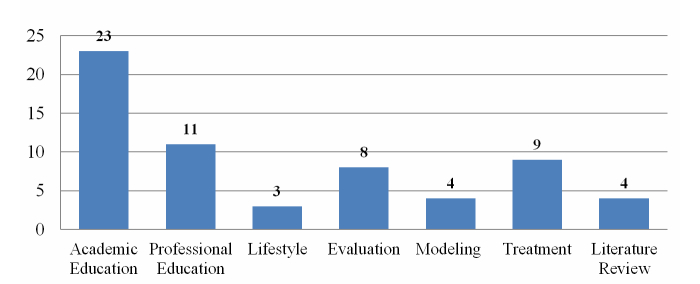
Number of published papers based on the categories.

In this study, we attempted to provide an overview of 3DVWs and their application areas in health care contexts. To this end, we found 62 different papers from five popular scientific databases. To gain a general understanding of 3DVW research, we classified these 62 research studies into six meaningful categories. Figure 6 shows the number of published studies in each category. The education category including professional and academic education contains the largest number of papers (n=34), of which 23 are related to the academic education category and 11 to the professional education category. In contrast, three papers are related to the lifestyle category, which has the least number of articles. In spite of the fact that treatment plays a crucial role in health care, only nine papers are allocated to this category. Eight papers have content related to assessment and evaluation, and in four of the papers the authors used 3DVWs as a platform for modeling various health-related environments. Of the total 62 papers, four papers included surveys and literature reviews on this subject.

3DVWs are increasing in popularity as a new medium for educational purposes, and pedagogical institutions are adopting this technology to support their teaching and learning. A wide range of well-known educational institutions around the world are using 3DVWs for various purposes, including distance education, presentations, meetings, and literature and language attainment. Since 3DVWs do not have a storyline or plot of avatars, actors, and events, the lack of a guiding narrative in these technologies provides flexibility for educators to design more complicated learning spaces for their pedagogical requirements. Not only is the technology propeling the use of 3DVWs in education and learning, but the new generation of students and learners is also demanding the use of this advanced technology in education. These are the main reasons that education, especially academic education, attracted more attention in the health care sector, compared to the other application areas.

### Implications for Researchers

In spite of the large number of studies on 3DVWs in health care, no systematic review has been conducted on this topic. There are several research papers that survey the range of health-related activities in virtual worlds [7-10]. For example, Kamel Boulos et al, in their hybrid review/case study, introduced 3DVWs and the educational potential of Second Life to medical/health librarians and educators. They described medical and health education examples from Second Life and compiled a companion resources page [73] with additional online material to support their study [7]. Hansen et al, in her review of existing literature, described the potential of health care learning environments in 3DVWs and provided relevant examples of 3DVWs [8]. Beard et al performed a literature survey and categorized the range of health-related activities in Second Life. They identified 68 health-related sites and examined the design attributes of the sites and assessed the utilities of Second Life for propagation of health information. They developed five categories to explain the range of online health-related activities [10]. But those literature reviews do not provide a comprehensive analysis of the topic, and their surveys were published between 2007 and 2010. There was no systematic review with a sufficient number of reviewed articles. In order to update the literature and provide a more comprehensive picture of 3DVW applications in health care, we decided to perform a systematic review examining 62 papers, a larger number than that covered by previous surveys and literature reviews. We also covered publications from January 1990 to July 2013.

The proposed taxonomy provides a big picture of the application areas of 3DVWs in health care. The most reviewed papers in this systematic review are in the academic and professional education categories—almost 54% of all papers (34/62). Despite the fact that researchers have paid more attention to education, there are gaps in this category. For example, studying the impact of 3DWVs in the education of surgeons could be an excellent starting point. Creating specific rooms and environments in virtual worlds so students can remotely access their course materials such as files, e-books, lecture captures, and presentation slides is a second suggestion. Training students and the public in first aid with 3DVWs is another.

Living a healthy lifestyle has become a common ambition, and people are now attempting to pay more attention to their health. There are, however, few studies in the literature regarding the impact of 3DVWs on people’s health and lifestyle. Therefore, investigating the advantages and disadvantages of applying this new technology in improving healthy behaviors and extending the health culture could be a useful endeavor.

Although there are studies related to treatment in health care and medicine in the current literature, there is a lack of research on some application areas of 3DVWs in this category. For example, in the treatment and psychology context, no study addresses problems such as social isolation, care of the elderly, phobias, and so on.

Existing studies pay little attention to the application of 3DVWs in modeling. According to our systematic review, few have been conducted in this field. Therefore, the powerful ability of these tools for modeling and simulating health scenarios could be investigated in greater depth. For example, investigating the impact of simulated sophisticated hospital equipment on the skill-building of hospital staff would be worth examining. Future research could also focus on replication of earlier studies with larger sample sizes to examine the impact of 3DVWs on various health care contexts.

### Implications for Practice

Our systematic review and the proposed taxonomy have various implications for a wide range of individuals and institutions that could use this study to recognize different areas of application of 3DVWs of benefit to their professional, business, and organizational decisions, for example, health decision-makers, universities, educational communities, hospitals, physicians and practitioners, nurses, patients, nursing homes, pharmaceutical institutes, health marketers, disaster managers, psychologists, public health providers, clinical medicine, addiction treatment institutes, etc.

Our systematic review shows that pedagogical uses are the most important applications of 3DVWs in health care. Universities, hospitals, health care organizations, training centers, educational institutions, and educators can use 3DVWs as a creative, powerful, and efficient tool for courses, lectures, and workshops. They can develop new effective ways of teaching and learning in virtual environments. Thanks to the Internet-based characteristics of these worlds, there is the possibility of distance learning; therefore, students, nurses, physicians, medical staff, and other trainees can connect to the specified worlds and attend a virtual classroom using their own computer. Teachers and trainers also have wider access to geographically remote trainees and distance learners.

Universities, colleges, hospitals, health care agencies, private groups, businesses, and corporations can use 3DVWs for discussion, seminars, presentations, meetings, and other similar activities by creating large virtual auditoriums and meeting rooms, and gathering their staff in specific environments within virtual worlds where digital materials can be created, stored, and used. These communities could also create virtual health and medical libraries to enable remote access to related e-books, documents, and other applications; 3DVWs have great potential for librarians and educators in remote locations.

We found that hospitals, universities, and medical training organizations can model laboratories, wards, and various parts of a hospital with instruments, equipment, and settings that simulate real-world activities at a very low cost for educational and pedagogical purposes. Virtual simulations in 3DVWs can be an acceptable strategy for delivering scenarios that focus on health-related skills. The critical life simulation enables educators to collaborate and solve problem-based scenarios in a team, allowing them to construct personal and technical skills actively through interaction in a virtual environment without the danger of making mistakes and risking harm to patients or themselves.

Our findings could benefit psychologists and psychological institutions with use of 3DVWs in their treatment and therapeutic measurements. These worlds could be an interesting tool for prevention and treatment of different psychological disorders. People with psychological and mental problems could benefit from using these worlds under the supervision of expert medical staff. Mental health specialists could devise specific remedial programs for patients suffering from psychological stress, anxiety, PTSD, schizophrenia, and other problems.

Addiction treatment institutes and therapeutic centres could use 3DVWs in their therapeutic programs to reduce the risk of abuse and addiction. Patients suffering from addiction disorders could receive behavioral treatments in virtual worlds that include the planning of specific ways to avoid addictive stimulus. By using therapeutic interventions in 3DVWs, addiction medicine specialists may be able to help patients learn healthier ways to find satisfaction.

Disaster managers could use 3DVWs for disaster preparedness and response training programs. By simulating similar disaster scenarios, they would be able to increase the experience, collaboration, leadership, disaster response, and decision-making skills of their personnel in various disaster scenarios.

Nursing homes and institutes with a responsibility for aged people might be able to use these worlds as an interesting tool for treatment as well as prevention of conditions such as social isolation. They could also be used to entertain people with physical disabilities and help them to cope with loneliness and social isolation.

It is clear that 3DVWs offer a wide range of features that could enhance marketing companies’ and health-related organizations’ marketing initiatives. Traditional advertising is currently replicated in VWs with digital billboard advertisements and product placement. Current and future real-life initiatives could also be replicated in these platforms as different simulations. Communities could use these platforms as a way of promoting hospitals, health care services, health system reform, and even fundraising.

Health decision-makers and policy-makers, health IT managers, health marketers, and medical business owners could use these results to find different areas of application of 3DVWs of benefit to their organizations. Furthermore, public health providers could focus on 3DVWs to extend public awareness and promote healthy lifestyles. For example, they could include videos, presentations, buildings, and even exercise and sport facilities in their private virtual islands to motivate users to adopt healthier behaviors in their daily life.

Patients with different kinds of social disorders and people who have problems communicating with others for different reasons could use 3DVWs to improve their social learning and interactive behaviors. A 3D virtual experience gives patients a feeling of control over their health, improving their knowledge and confidence, since they can navigate the health care system from their own home.

Physicians and practitioners can be aware of the latest virtual facilities in different 3DVWs and apply them in their therapeutic and medical treatments. They can create specific environments to meet their clinical needs as well as the needs of their patients. For example, they can communicate with their patients through these platforms to perform check-ups and issue prescriptions and necessary guidance.

3DVWs play a crucial role in the assessment and evaluation of diverse skills in health care. For example, virtual patients can be used by trainees such as nurses, surgeons, students, and other medical staff, and their performance can be assessed and benchmarked in different ways.

### Limitations

The quality of these results is highly dependent on the quality of the papers that have been identified. Therefore, it is not possible to evaluate the quality of the results in this study. We cannot guarantee that we have taken all relevant applications of 3DVWs in health care into account because there are limited studies related to this topic. Any systematic review is limited to its keywords; this study is no exception. We cannot present any global conclusions about the application areas of 3DVWs in health care because only a few of the studies were conducted in countries other than the United States and the United Kingdom.

### Conclusions

This study provides a comprehensive picture of 3DVW’s application in health care and updates the literature in this field. It also highlights various health contexts and subcontexts that have applied 3DVWs. The results of this systematic literature review could be beneficial for researchers interested in this topic to better understand the field and previous studies, better classify research, help them shape the future direction of research, and identify gaps in the literature. By updating work in the application areas of 3DVWs, this study also attempts to help a wide variety of individuals and organizations, such as practitioners, nurses, managers, hospitals, health care agencies, private groups, business health companies and corporations, and universities to recognize various areas of application of 3DVWs and determine directions for practice in their own areas.

To conclude, it is evident that 3D virtual worlds present several innovative ways to carry out health-related activities. In this study, we developed six main categories to explain the application areas of 3DVWs in various health care contexts: academic education, professional education, treatment, modeling, lifestyle, and evaluation. Our proposed taxonomy could be used to provide an overview of the application of 3DVWs in health care and medical research and practice that individuals, professional health communities, and academic institutions could use in their various activities.

## Multimedia Appendix 1

Search terms used in each database.

## Abbreviations

3D: three dimensional
3DVW: three-dimensional virtual world
CPR: cardiopulmonary resuscitation
PTSD: posttraumatic stress disorder
RFID: radio-frequency identification
RSI: rapid sequence intubation
TAM: technology acceptance model
VR: virtual reality
VW: virtual world
