# Supplementary material for: A Decade of Research on the Use of Three-Dimensional Virtual Worlds in Health Care: A Systematic Literature Review
Source: J Med Internet Res. 2014 Feb 18;16(2):e47. doi: 10.2196/jmir.3097 (PMC3958677; doi:10.2196/jmir.3097)
Supplement: Supplementary file 1 [file jmir_v16i2e47_app1.pdf]

Multimedia Appendix 1. Search terms used in each database.

Scientific database	Search option	Search term
ScienceDirect online database	Expert search	TITLE-ABSTR-KEY("3D" OR "3 D" OR "3-D" OR "3_D" OR "three-dimensional" OR "three dimensional" OR "3 dimensional" OR "three D" OR "three-D") AND TITLE-ABSTR-KEY("virtual world" OR "virtual life" OR "virtual space" OR "virtual environment" OR "virtual reality" OR "virtual community" OR "social media" OR "social network" OR "cyberspace" OR "simulated environment" OR "virtual inhabited world" OR "inhabited space" OR "second life" OR "active world" OR "avatar") AND TITLE-ABSTR-KEY("health" OR "medical" OR "medicine" OR "clinical" OR "nursing" OR "illness" OR "disease" OR "diagnosis" OR "care" OR "pharmacy" OR "nutrition" OR "physician" OR "doctor" OR "hospital" OR "surgical" OR "EMR" OR "EHR" OR "HIS").
ProQuest Computing	Command line search	all("3D" OR "3 D" OR "3-D" OR "3_D" OR "three-dimensional" OR "three dimensional" OR "3 dimensional" OR "three D" OR "three-D") AND all("virtual world" OR "virtual life" OR "virtual space" OR "virtual environment" OR "virtual reality" OR "virtual community" OR "social media" OR "social network" OR "cyberspace" OR "simulated environment" OR "virtual inhabited world" OR "inhabited space" OR "second life" OR "active world" OR "avatar") AND all("health" OR "medical" OR "medicine" OR "clinical" OR "nursing" OR "illness" OR "disease" OR "diagnosis" OR "care" OR "pharmacy" OR "nutrition" OR "physician" OR "doctor" OR "hospital" OR "surgical" OR "EMR" OR "EHR" OR "HIS")
PubMed	PubMed advanced search builder	(("3D"[Title/Abstract]) OR ("3 D"[Title/Abstract]) OR ("3-D"[Title/Abstract]) OR ("3_D"[Title/Abstract]) OR ("three-dimensional"[Title/Abstract]) OR ("three dimensional"[Title/Abstract]) OR ("3 dimensional"[Title/Abstract]) OR ("three D"[Title/Abstract]) OR ("three-D"[Title/Abstract])) AND (("virtual world"[Title/Abstract]) OR ("virtual life"[Title/Abstract]) OR ("virtual space"[Title/Abstract]) OR ("virtual environment"[Title/Abstract]) OR ("virtual reality"[Title/Abstract]) OR ("virtual community"[Title/Abstract]) OR ("social media"[Title/Abstract]) OR ("social network"[Title/Abstract]) OR ("cyberspace"[Title/Abstract]) OR ("simulated environment"[Title/Abstract]) OR ("virtual inhabited world"[Title/Abstract]) OR ("inhabited space"[Title/Abstract]) OR ("second life"[Title/Abstract]) OR ("active world"[Title/Abstract]) OR ("avatar"[Title/Abstract])) AND (("health"[Title/Abstract]) OR ("medical"[Title/Abstract]) OR ("medicine"[Title/Abstract]) OR ("clinical"[Title/Abstract]) OR ("nursing"[Title/Abstract]) OR ("illness"[Title/Abstract]) OR ("disease"[Title/Abstract]) OR ("diagnosis"[Title/Abstract]) OR ("care"[Title/Abstract]) OR ("pharmacy"[Title/Abstract]) OR ("nutrition"[Title/Abstract]) OR ("physician"[Title/Abstract]) OR ("doctor"[Title/Abstract]) OR ("hospital"[Title/Abstract]) OR ("surgical"[Title/Abstract]) OR ("EMR"[Title/Abstract]) OR ("EHR"[Title/Abstract]) OR ("HIS"[Title/Abstract])) AND ("1990"[Date - Publication] : "2013"[Date - Publication]))
IEEE Xplore	Command search	("Abstract":"3D" OR "Abstract":"3 D" OR "Abstract":"3-D" OR "Abstract":"3_D" OR "Abstract":"three-dimensional" OR "Abstract":"three dimensional" OR "Abstract":"3 dimensional" OR "Abstract":"three D" OR "Abstract":"three-D") AND ("Abstract":"virtual world" OR "Abstract":"virtual life" OR "Abstract":"virtual space" OR "Abstract":"virtual environment" OR "Abstract":"virtual reality" OR "Abstract":"virtual community" OR "Abstract":"social media" OR "Abstract":"social network" OR "Abstract":"cyberspace" OR "Abstract":"simulated environment" OR "Abstract":"virtual inhabited world" OR "Abstract":"inhabited space" OR "Abstract":"second life" OR "Abstract":"active world" OR "Abstract":"avatar") AND ("Abstract":"health" OR "Abstract":"medical" OR "Abstract":"medicine" OR "Abstract":"clinical" OR "Abstract":"nursing" OR "Abstract":"illness" OR "Abstract":"disease" OR "Abstract":"diagnosis" OR "Abstract":"care" OR

		"Abstract":"pharmacy" OR "Abstract":"nutrition" OR "Abstract":"physician" OR "Abstract":"doctor" OR "Abstract":"hospital" OR "Abstract":"surgical" OR "Abstract":"EMR" OR "Abstract":"EHR" OR "Abstract":"HIS")
ACM Digital Library	Advanced search	("Abstract":"3D" OR "Abstract":"3 D" OR "Abstract":"3-D" OR "Abstract":"3_D" OR "Abstract":"three-dimensional" OR "Abstract":"three dimensional" OR "Abstract":"3 dimensional" OR "Abstract":"three D" OR "Abstract":"three-D") AND ("Abstract":"virtual world" OR "Abstract":"virtual life" OR "Abstract":"virtual space" OR "Abstract":"virtual environment" OR "Abstract":"virtual reality" OR "Abstract":"virtual community" OR "Abstract":"social media" OR "Abstract":"social network" OR "Abstract":"cyberspace" OR "Abstract":"simulated environment" OR "Abstract":"virtual inhabited world" OR "Abstract":"inhabited space" OR "Abstract":"second life" OR "Abstract":"active world" OR "Abstract":"avatar") AND ("Abstract":"health" OR "Abstract":"medical" OR "Abstract":"medicine" OR "Abstract":"clinical" OR "Abstract":"nursing" OR "Abstract":"illness" OR "Abstract":"disease" OR "Abstract":"diagnosis" OR "Abstract":"care" OR "Abstract":"pharmacy" OR "Abstract":"nutrition" OR "Abstract":"physician" OR "Abstract":"doctor" OR "Abstract":"hospital" OR "Abstract":"surgical" OR "Abstract":"EMR" OR "Abstract":"EHR" OR "Abstract":"HIS")
